# Different Requirement for Wnt/β-Catenin Signaling in Limb Regeneration of Larval and Adult *Xenopus*

**DOI:** 10.1371/journal.pone.0021721

**Published:** 2011-07-26

**Authors:** Hitoshi Yokoyama, Tamae Maruoka, Haruki Ochi, Akio Aruga, Shiro Ohgo, Hajime Ogino, Koji Tamura

**Affiliations:** 1 Department of Developmental Biology and Neurosciences, Graduate School of Life Sciences, Tohoku University, Aoba-ku, Sendai, Japan; 2 Graduate School of Biological Sciences, Nara Institute of Science and Technology (NAIST), Takayama, Ikoma, Nara, Japan; 3 JST, CREST, 5, Sanbancho, Chiyoda-ku, Tokyo, Japan; University of Colorado, Boulder, United States of America

## Abstract

**Background:**

In limb regeneration of amphibians, the early steps leading to blastema formation are critical for the success of regeneration, and the initiation of regeneration in an adult limb requires the presence of nerves. *Xenopus laevis* tadpoles can completely regenerate an amputated limb at the early limb bud stage, and the metamorphosed young adult also regenerates a limb by a nerve-dependent process that results in a spike-like structure. Blockage of Wnt/β-catenin signaling inhibits the initiation of tadpole limb regeneration, but it remains unclear whether limb regeneration in young adults also requires Wnt/β-catenin signaling.

**Methodology/Principal Findings:**

We expressed heat-shock-inducible (hs) Dkk1, a Wnt antagonist, in transgenic *Xenopus* to block Wnt/β-catenin signaling during forelimb regeneration in young adults. hsDkk1 did not inhibit limb regeneration in any of the young adult frogs, though it suppressed Wnt-dependent expression of genes (*fgf-8* and *cyclin D1*). When nerve supply to the limbs was partially removed, however, hsDkk1 expression blocked limb regeneration in young adult frogs. Conversely, activation of Wnt/β-catenin signaling by a GSK-3 inhibitor rescued failure of limb-spike regeneration in young adult frogs after total removal of nerve supply.

**Conclusions/Significance:**

In contrast to its essential role in tadpole limb regeneration, our results suggest that Wnt/β-catenin signaling is not absolutely essential for limb regeneration in young adults. The different requirement for Wnt/β-catenin signaling in tadpoles and young adults appears to be due to the projection of nerve axons into the limb field. Our observations suggest that nerve-derived signals and Wnt/β-catenin signaling have redundant roles in the initiation of limb regeneration. Our results demonstrate for the first time the different mechanisms of limb regeneration initiation in limb buds (tadpoles) and developed limbs (young adults) with reference to nerve-derived signals and Wnt/β-catenin signaling.

## Introduction

Limb regeneration in amphibians is one of the most fascinating examples of organ or appendage regeneration among tetrapods. While all tetrapod limbs are homologous, i.e., derived from common ancestral pairs of fish fins [1], the ability of limbs to regenerate varies greatly among tetrapod classes [2]. Anuran (frog) tadpoles and urodeles (newts and salamanders) are the only tetrapods that can fully regenerate amputated limbs. In the case of anuran amphibians, such as *Xenopus laevis*, the tadpole can completely regenerate its developing hindlimb buds prior to the onset of metamorphosis, but the regenerative ability declines gradually as metamorphosis proceeds [3], [4]. A young post-metamorphosis adult, the *Xenopus* froglet, regenerates only an unbranched cartilaginous spike-like structure after limb amputation ([3]; reviewed in [5]).

Epimorphic regeneration, including limb regeneration, always proceeds by local formation of a “regeneration blastema,” a growth zone of mesenchymal stem/progenitor cells, on the stump. After amputation, limb regeneration in amphibians progresses through a characteristic series of steps, beginning with wound healing, followed by formation of the blastema, and finally by redevelopment (reviewed in [6], [7], [8], [9]). Although the redevelopment stage of limb regeneration seems equivalent to limb development, the early steps leading to genesis of the blastema are critical in determining whether or not an amputated limb can successfully regenerate. Once a blastema is successfully formed, it can regenerate autonomously as a self-organizing system ([10], [11]; reviewed in [12], [13]). Therefore, it is possible that elucidation of the critical factor(s) for blastema formation in the early stage of amphibian limb regeneration will enable us to control limb regenerative ability and will ultimately contribute to organ replacement therapy [12], [14].

Classic experiments suggested that signals from nerves are essential for the initiation of limb regeneration. It is well known that limb regeneration of amphibians is dependent on substances released by nerves (e.g., growth factors), once the limb region is substantially innervated (reviewed in [15]). If the nerve trunks are removed from the limb stump, the denervated stump fails to regenerate. However, if denervation is performed after a certain stage of blastema formation (medium bud stage), limb regeneration is not blocked, but regenerate is small [16]. Conversely, ectopic nerve deviation to a wound on the side of a limb can induce a blastema-like outgrowth (bump) in urodeles ([17], [18]; refined in [19]). There are several candidates for these nerve-derived signals, including FGF-2 [20], GGF [21], [22], and nAG [23]. Here, the term, “nerve signals,” refers to such substances and not to the electric signals that are transmitted chemically across synapses.

The difficulty in manipulating gene function in postembryonic amphibians has hindered functional analysis of the genes and signaling pathways that might participate in regeneration. However, the development of efficient transgenic systems in *Xenopus* has enabled manipulation of gene expression in postembryonic amphibians (e.g., [24], [25], [26]). For limb regeneration, two major signaling pathways, BMP [27] and Wnt/β-catenin [28], have been shown to be essential in transgenic *Xenopus* in which the expression of noggin, a BMP antagonist, or Dkk1, a Wnt/β-catenin antagonist, is induced under the control of a heat-shock promoter (hsp70) (reviewed in [9], [29], [30]). When either of these signaling pathways was temporarily inhibited by one or two heat-shocks early in the regeneration process, regeneration of tadpole limb buds was blocked. Therefore, morphogenic signaling pathways (BMP and Wnt/β-catenin) and nerve signals are both thought to play essential roles in the initiation of limb regeneration, but their relative contributions and the relationships among these signals remain unclear.

The expression of an ectopic BMP or Wnt/β-catenin antagonist effectively blocks regeneration when the paddle-shaped limb bud of a tadpole is amputated [27], [28]. However, this early-stage limb bud is not yet heavily innervated, and its regeneration does not require nerve signals, although a limb bud acquires a “nerve dependency” for limb regeneration at later stages, after it is heavily innervated [31], [32]. It is unclear whether Wnt/β-catenin (and BMP) signaling is still essential for the initiation of limb regeneration in late-stage tadpoles or metamorphosed young adults (froglets), in which the limbs cannot regenerate without nerve signals.

In this study, we assessed the role of Wnt/β-catenin signaling in froglet limb regeneration by preparing heat-shock-inducible Dkk1 (hsDkk1) transgenic froglets. In contrast to the previously reported essential role for Wnt/β-catenin signaling in tadpole regeneration, hsDkk1 did not interfere with regeneration in the froglet (spike regeneration), although it did suppress downstream targets of Wnt/β-catenin signaling. Therefore, Wnt/β-catenin signaling appears to be dispensable for the regeneration of froglet limbs. However, when a froglet limb was partially denervated, hsDkk1 did have an inhibitory effect on its regeneration. These results suggest that Wnt/β-catenin signaling is differently involved in limb regeneration in tadpoles and froglets and suggest that nerve signals can substitute for Wnt/β-catenin signaling in limb regeneration.

## Results

### *Wnt-3a* is expressed in the froglet blastema

**Figure 1:**
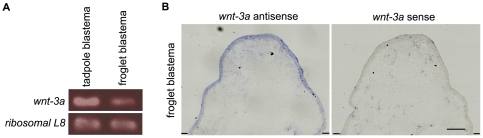
*Wnt-3a* is expressed in the blastema of both froglets and tadpoles. (A) RT-PCR of the total RNA from tadpole blastemas at 5 dpa or froglet blastemas at 9 dpa showed that_*wnt-3a* was expressed in both the tadpole and froglet blastemas. (B) In situ hybridization of froglet blastemas at 9 dpa with a *wnt-3a* antisense probe revealed expression of *wnt-3a* in the epidermal layer of the blastema, while no specific signal was detected with the *wnt-3a* sense probe. Each section was hybridized with the antisense or sense probe, at the same time and by the same procedure, and the development of the staining reaction was stopped at exactly the same time. The lines indicate the estimated amputation plane. Scale bar = 100 µm.

In most experiments, we used forelimbs to analyze froglet limb regeneration because hindlimbs are essential for swimming, and hindlimb amputation can result in drowning or exsanguination of the animal (unpublished observation). Froglet forelimbs and hindlimbs regenerate the same spike-like structure [33], [34]. In the chick embryo, *wnt-3a* is expressed in ectodermal cell layers during formation of the apical ectodermal ridge (AER), a specialized ectodermal structure essential for the outgrowth of amniote limb buds, and induces *fgf-8* expression in a β–catenin-dependent manner [35]. Previous studies showed that *wnt-3a* is expressed in the apical epidermis of the blastema of tadpoles amputated at stage 52 [36] and that *wnt-3a* expression overlaps with *fgf-8* expression [28]. RT-PCR analysis showed that *wnt-3a* was expressed in cone-shaped blastemas of both froglets and tadpoles (Figure 1A). We then examined *wnt-3a* expression by in situ hybridization of sectioned froglet blastemas and found that it was expressed in the apical epidermis of the cone-shaped blastema at 9 days post-amputation (dpa) (Figure 1B). While the expression domain of *wnt-3a* is broader than that of *fgf-8*, the two domains overlap ([33], [37] and data not shown) in the froglet blastema and in the tadpole blastema [28]. The similar expression patterns of *wnt-3a* in froglet and tadpole blastemas raises the possibility that Wnt/β-catenin signaling has an essential role in limb regeneration of froglets as well as tadpoles.

### Expression of a modified hsDkk1GFP construct in *Xenopus*

**Figure 2:**
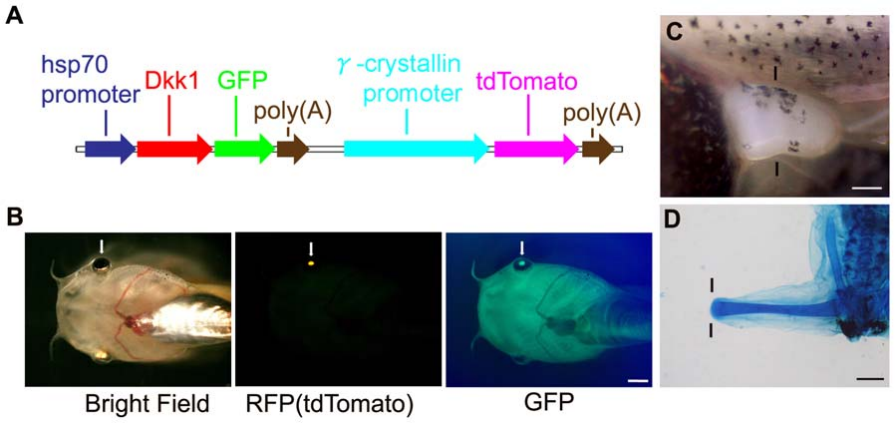
Refined procedure for heat-shock-inducible inhibition of Wnt/β-catenin signaling in *Xenopus laevis*. (A) Map of the heat-shock-inducible Dkk1GFP transgene with lens labeling. Details are described in Experimental Procedures. The bright RFP variant, tdTomato under control of the 2.2-kb *γ–crystallin* promoter, was introduced downstream of hsp70-Dkk1GFP. (B) Prior to heatshock, a tadpole containing the transgene could be recognized by the tdTomato fluorescence in its lenses. After heatshock, ubiquitous Dkk1GFP expression was induced in the lens-labeled tadpoles. Note that the tdTomato protein in the lens was detected through a GFP filter as well as through an RFP filter. (C) Dorsal view of the left hindlimb bud at stage 52. (D) Induction of hsDkk1 expression at stage 52 completely blocked regeneration of the hindlimb bud. Nothing was regenerated from the amputation level. Lines indicate the estimated amputation planes (knee level for the hindlimb bud). Arrows indicate the lens region of tadpoles. Scale bar = 1 mm for (B) and (D) and 250 m for (C). See also Figure S1.

With the original construct, Hsp70-Dkk1GFP [28], we cannot predict which transgenic F0 individuals contain the transgene prior to heat shock. To improve the efficiency of heat-shock-inducible gene manipulation, we modified the Hsp70-Dkk1GFP (hsDkk1GFP) construct by placing the gene for tdTomato under control of the gamma crystallin promoter (Figure 2A). Using this construct, we could identify F0 individuals containing the transgene by the red fluorescence of tdTomato in the lens region, prior to performing the heat-shock (Figure 2B, middle). Within 3 to 4 hours after heatshock, hsDkk1GFP fluorescence could be detected in most individuals that showed tdTomato fluorescence in the lens (Figure 2B, right). However, hsDkk1 expression sometimes could not be detected in these tdTomato-positive animals, and hsDkk1 expression was induced by heatshock in a few individuals lacking tdTomato fluorescence in the lens. These unusual transgenic individuals were excluded from the following experiments. Because of the random insertion of transgenes into *Xenopus* genomes by the REMI transgenic procedure [38], a considerable percentage of F0 animals did not show tdTomato fluorescence in the lens or induction of hsDkk1 expression by heat-shock and they were therefore used as matched sibling negative controls (wild-type).

It was previously reported that the regeneration of limb buds amputated at the young tadpole stage (stage 52; Figure 2C) is blocked by hsDkk1 expression, while wild-type limb buds can regenerate well [28]. When tadpoles with our modified hsDkk1GFP construct were heat-shocked at stage 52 and their hindlimb buds were amputated 3 to 4 h later, limb regeneration was effectively blocked (Figure 2D, Table 1). This result indicated that the modified hsDkk1GFP construct, with the gene for tdTomato under control of the γ–crystallin promoter, exerted the same effect as that of the original hsDkk1GFP construct. We therefore used this modified construct in the following experiments to efficiently select transgenic (tg) individuals that were positive for hsDkk1.

**Table 1:** Regenerative capacity of tadpole hindlimb buds heat-shocked and amputated at stage 52.

Type of tadpole (wild-type or hsDkk1)	Total number of limb buds	No regeneration occured	Some regeneration occurred					
			Incomplete<------------------------------------------------------------------------------------>					complete
		**none**	**1 spike**	**1 digit**	**2 digits**	**3 digits**	**4 digits**	**5 digits**
**wild-type**	10	4	0	2	0	0	0	4
**Dkk1GFP**	11	11	0	0	0	0	0	0

To exclude the possibility that Wnt/β-catenin signaling has a different role in regeneration of the forelimb bud vs. the hindlimb bud, we repeated our experiment by expressing hsDkk1 during forelimb bud regeneration in tadpoles. At stage 54, a forelimb bud becomes paddle-shaped, comparable to the hindlimb bud at stage 52 (Figure S1A, [39]). Regeneration of forelimb buds amputated at stage 54 was efficiently blocked in the hsDkk1 tadpoles by heatshock given 3 to 4 h prior to amputation (Figure S1C and Table S1). Therefore, Wnt/β-catenin is required for the initiation of both forelimb and hindlimb bud regeneration in tadpoles.

### Different contributions of Wnt/β-catenin signaling to limb regeneration of tadpoles versus froglets

**Figure 3:**
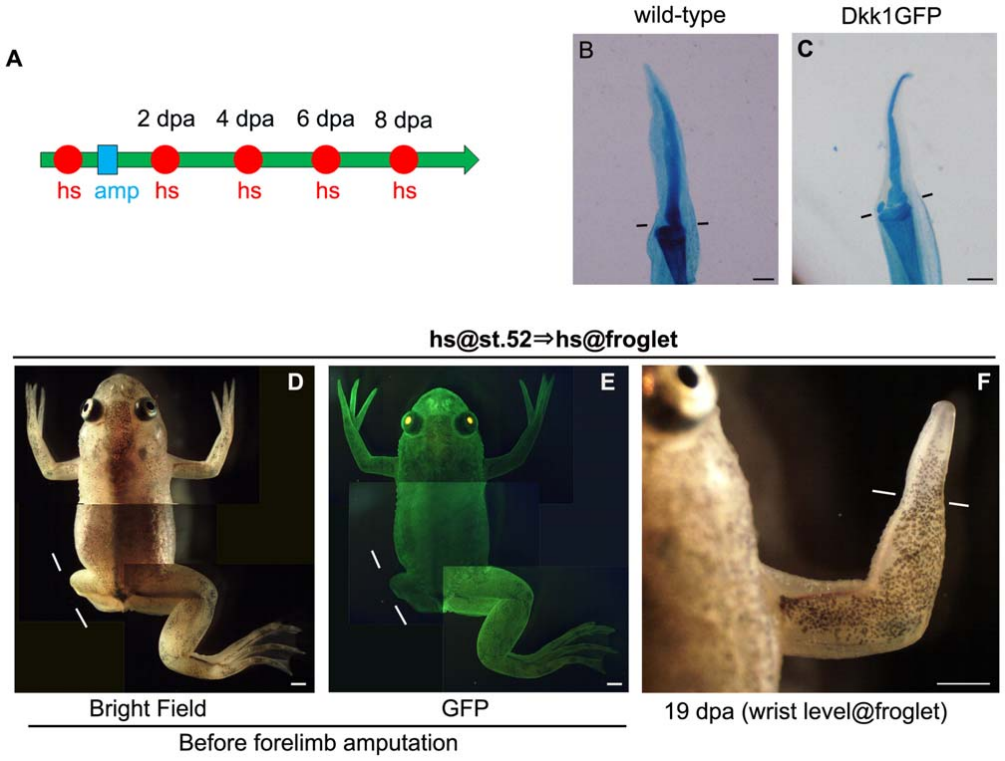
hsDkk1 does not block spike regeneration in the froglet. (A) Experimental scheme for froglets. One heatshock (hs: represented as a red circle) was applied to the froglets 3 to 4 h before amputation. The forelimb was amputated through the distal zeugopodium (amp: represented as a blue square) followed by a heatshock every other day until 8 dpa. (B and C) The hsDkk1 froglets (C) regenerated a spike like the wild-type froglets (B). (D-F) hsDkk1 had a different effect on regenerative capacity of the tadpole and froglet within the same individual. hsDkk1 expression induced at the tadpole stage (st. 52) blocked regeneration of the left hindlimb bud amputated at the presumptive knee level (D). After this hsDkk1 tadpole had become a froglet, hsDkk1GFP expression could still be induced effectively by heatshock (E). However, repeated heatshocks as shown in (A) could not block forelimb regeneration of the same hsDkk1 froglet (F). Lines indicate the estimated amputation plane (knee level for the hindlimb bud, distal zeugopod level for the forelimb). Scale bar = 500 µm for (B) and (C) and 1 mm for (D), (E), and (F).

We raised the hsDkk1 tg tadpoles to the froglet stage and assessed the Wnt requirement for spike regeneration. To block Wnt/β-catenin signaling, the hsDkk1 froglets were heat-shocked and forelimbs were amputated through the distal zeugopod 3 to 4 hours later. As seen at the tadpole stage, hsDkk1GFP expression was induced after heatshock in the majority of hsDkk1 froglets that showed tdTomato fluorescence in their lenses (Figure 3E). As with the tadpoles, we excluded froglets that expressed either tdTomato in the absence of hsDkk1 or vice versa from this experiment and further experiments. In contrast to the hsDkk1 tg tadpoles, the hsDkk1 tg froglets showed spike regeneration even though the GFP fluorescence after heatshock was as bright as that in the tadpoles (data not shown, see also Figure 3E).

The fluorescence of Dkk1GFP peaks one day after heatshock, persists for several days, and then diminishes [28]. Because the formation of a cone-shaped blastema takes one week or longer in froglets, it was possible that one heatshock was insufficient to suppress Wnt/β-catenin signaling during the initiation of limb regeneration. We therefore performed 5 heatshocks 3–4 hours before amputation and then performed heatshock every other day until 8 dpa (Figure 3A), when formation of the cone-shaped blastema is usually completed. Although it was expected that Wnt/β-catenin signaling would be blocked during the initiation of limb regeneration in these repeatedly heat-shocked froglets, all of the froglets still regenerated a spike, like the wild-type controls (Figure 3B and 3C; Table 2). Therefore, in contrast to tadpoles, Wnt/β-catenin signaling appears to be dispensable for the initiation of limb regeneration in froglets.

**Table 2:** Regenerative capacity of froglet forelimbs repeatedly heat-shocked by 8 dpa.

Type of froglets (wild-type or hsDkk1)	Total number of forelimbs	No regeneration occurred	Some regeneration occurred
		**none**	**1 spike**
**wild-type**	13	0	13
**Dkk1GFP**	12	0	12
**wild-type (half-denervated)**	13	2	11
**Dkk1GFP (half-denervated)**	15	10	5

We heat-shocked tg individuals at the tadpole stage (stage 52) and again after metamorphosis to compare the Wnt requirement for limb regeneration in the same animal at both stages. For this experiment, hsDkk1 tg tadpoles were heat-shocked once at stage 52 and one of their hindlimb buds was amputated after the hsDkk1GFP fluorescence was clearly visible. Regeneration of the hindlimb bud was blocked, while the nonamputated forelimb and hindlimb buds developed normally (Figure 3D and 3E, n = 2). In these tg individuals, therefore, Wnt/β-catenin signaling was thought to be effectively blocked by heatshock through induced hsDkk1 expression. We raised these tg individuals to the froglet stage and repeated the heatshocks and forelimb amputation, as shown in Figure 3A. Even though regeneration at the hindlimb bud was effectively blocked by hsDkk1 at the tadpole stage, and bright GFP fluorescence was induced in the heat-shocked froglets (Figure 3E), the spike regeneration in these individuals was not blocked by the repeated heatshocks (Figure 3F, n = 2). These results indicating different regenerative responses within the same tg animals support the idea that Wnt/β-catenin signaling is dispensable for limb regeneration in froglets.

### Histological and gene expression analyses indicate different roles for Wnt/β-catenin signaling in limb regeneration intadpoles and froglets

**Figure 4:**
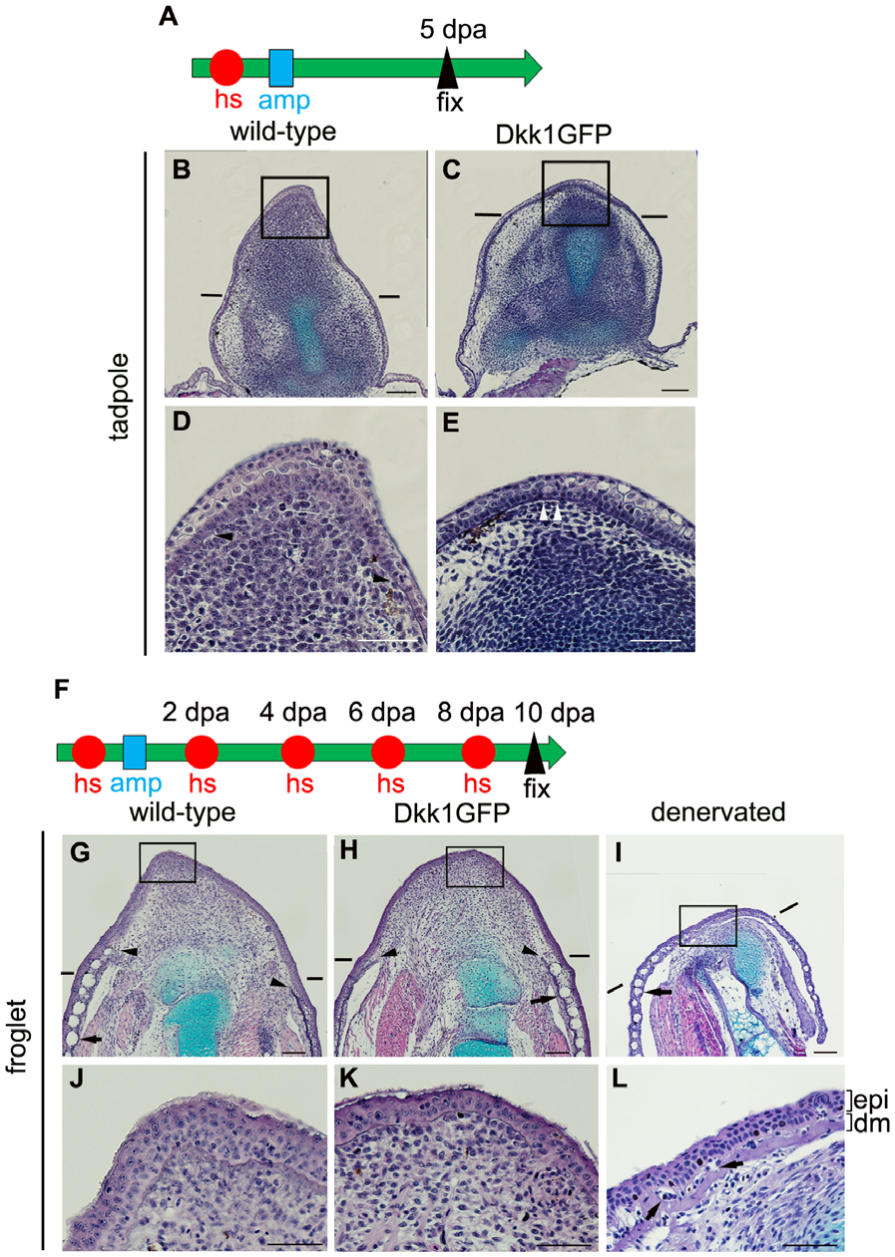
Histological examination of limb blastemas/stumps with or without blockage of Wnt/β-catenin signaling. (A) Experimental scheme for tadpoles. One heatshock (hs: represented as a red circle) was applied to stage 52 tadpoles 3 to 4 h before amputation. The hindlimb buds were amputated at the presumptive knee level (amp: represented as a blue square), and fixation was done at 5 dpa. (B–E) Longitudinal sections of the limb blastema/stump of wild-type (B, D) and Dkk1GFP (C, E) tadpoles. Panels D and E are high-power views of the boxed regions in B and C, respectively. The distinct basement membrane is indicated by arrowheads. No distinct basement membrane was seen at the tip of the blastema, indicated by arrowheads in (D), while the basement membrane covered the entire amputation plane of the limb stump in (E). (F) Experimental scheme for froglets. One heatshock was applied 3 to 4 h before amputation. The forelimbs of the froglets were amputated through the distal zeugopodium followed by a heatshock every other day, and fixation was done at 10 dpa. (G–L) Longitudinal sections of the limb blastema/stump of wild-type (G, J) and Dkk1GFP (H, K) froglets. Panels J and K are high-power views of the boxed regions in G and H, respectively. Cone-shaped blastemas were formed in both wild-type (G) and Dkk1GFP (H) froglets. These blastemas were covered with dermis-free epidermis, and no skin gland was seen in the blastemal region of wild type (J) or Dkk1GFP froglets (K). In the denervated forelimb, no cone-shaped blastema was formed (I) and a differentiated dermis with skin glands covered the amputation plane at 10 dpa (L). ep, epidermis; dm, dermis. Arrows indicate skin glands. Arrowheads indicate the edge of a distinct basement membrane. Lines indicate the estimated amputation planes. Scale bar = 100 µm for (B), (C), (G), (H), and (I) and 50 µm for (D), (E), (J), (K), and (L).

We performed histological staining to examine in detail the different effects of hsDkk1 on limb regeneration in tadpoles and froglets. Paraffin sections of limb stumps and blastemas were stained with hematoxylin, eosin (HE), and Alcian blue. At the tadpole stage, histological morphology was clearly different in wild-type and hsDkk1 tissues. In wild-type controls, the cone-shaped blastema did not have a distinct basement membrane beneath the apical epidermis (Figure 4B and 4D). In contrast, in the hsDkk1 tadpoles, a degenerated (flattened) blastema-like structure formed, and a distinct basement membrane was visible throughout the amputation plane (Figure 4C and 4E).

In the froglets, unlike the tadpoles, there was no clear difference between histological morphology of wild-type blastemas and that of hsDkk1 blastemas. Both the hsDkk1 tg froglets and wild-type controls had formed a cone-shaped blastema at 10 dpa, with a dermis-free area underneath the apical epidermis (Figure 4G, 4H, 4J, and 4K). When the nerve trunks were removed from the limb stump, no cone-shaped blastema formed, and a differentiated dermal layer containing skin glands formed by 10 dpa between the overlying epidermis and the cartilage of the stump, as previously reported (Figure 4I and 4L, denervated) [33], [37]. These observations supported the idea that hsDkk1 interferes with limb regeneration of tadpoles but not that of froglets.

For further confirmation that the Wnt/β-catenin signaling was effectively blocked in the froglets, we next examined the expression of *fgf-8* as an index of Wnt/β-catenin activity in limbs during morphogenesis. Several studies have suggested that Wnt/β-catenin signaling controls the expression of *fgf-8* in the developing limb buds of the chick and mouse ([35], [40], [41]; reviewed in [42]). Moreover, defects in Wnt/β-catenin signaling caused reduction of Wnt/β-catenin responsive reporter activity as well as the absence of *fgf-8* expression in the apical epidermis of the mouse embryo [43]. A previous work in *Xenopus* also showed that *fgf-8* expression is quickly suppressed in the blastema of hsDkk1 tadpoles after heatshock [28].

**Figure 5:**
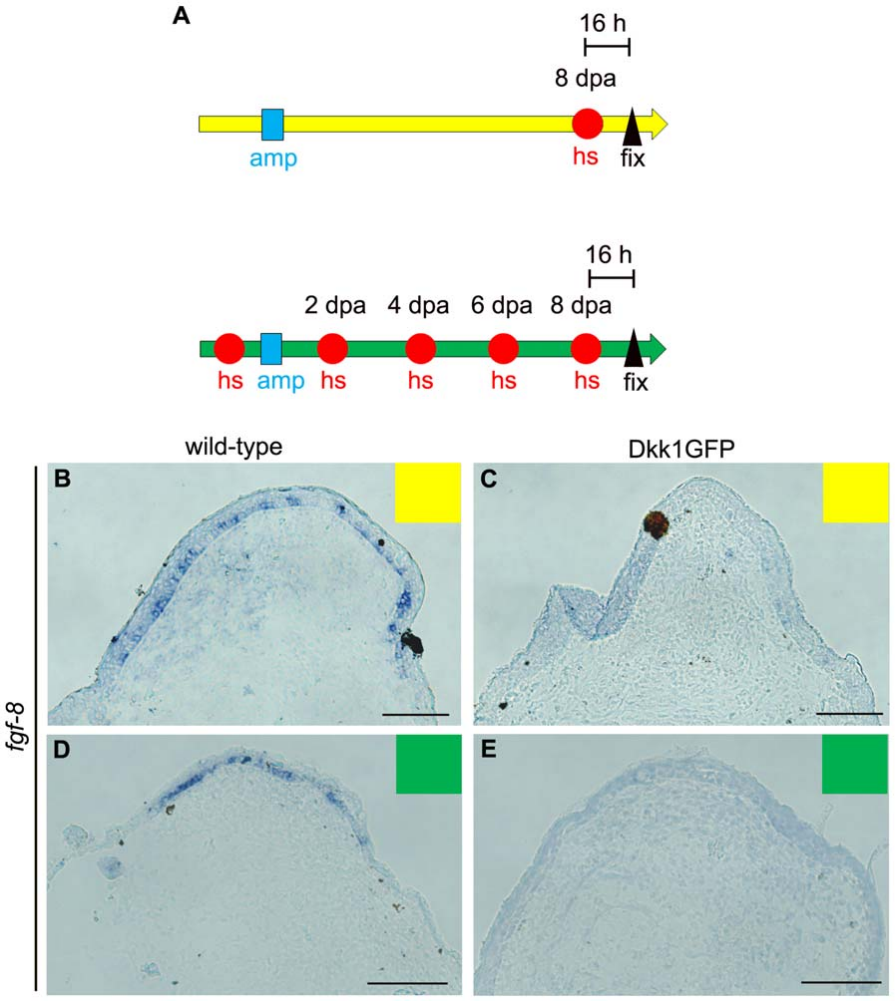
Wnt/β-catenin signaling is blocked in hsDkk1 froglets. (A) Experimental scheme for in situ hybridization. Upper: Froglet forelimbs were amputated (amp: blue square) and heat-shocked (hs: red circle) at 8 dpa, and their blastemas were excised and fixed (fix: black triangle) 16 h after the heatshock for in situ hybridization. Lower: Froglet forelimbs were amputated (amp: blue square) repeatedly heat-shocked (hs: red circles) every other day until 8 dpa, and their blastemas were excised and fixed (fix: black triangle) 16 h after the last heatshock for in situ hybridization. (B and C) In situ hybridization on sectioned samples of froglet blastemas that had been heat-shocked as shown in the upper scheme in (A). The sectioned samples were hybridized with the *fgf-8* antisense probe. (D and E) In situ hybridization of sectioned samples of froglet blastemas that were repeatedly heat-shocked as shown in the lower scheme in (A). Sectioned samples were hybridized with the *fgf-8* antisense probe. To guarantee correct comparisons of gene expression levels, wild-type (B or D) and hsDkk1GFP froglet (C or E) sections were treated in exactly the same way. Scale bar = 100 µm.

We therefore compared the expression levels of *fgf-8* in hsDkk1 and wild-type controls by in situ hybridization, as previously reported for tadpoles [28]. The froglets were heat-shocked only once, at 8 dpa (Figure 5A, upper), and their blastemas were then fixed. Alternatively, the froglets were repeatedly heat-shocked every other day until 8 dpa, as in the experiments for which results are shown in Figure 3 and Figure 4, and their blastemas were then fixed (Figure 5A, lower). The expression of *fgf-8* was clearly suppressed in the blastemas of the hsDkk1 froglets after repeated heatshocks (Figure 5E; n = 3/3) or even after a single heatshock (Figure 5C; n = 3/3). In contrast, in control sections, *fgf-8* was detected in the inner layer of the apical epidermis, as previously shown (Figure 5D and 5B; n = 3/3 for each course of heatshock; [33], [37]). We also quantified the expression level of *cyclin D1*, which is broadly expressed in the limb mesenchyme [44] and is a well-studied direct target of the Wnt/β-catenin pathway [45], [46], [47]. Quantitative RT-PCR analysis indicated that the amount of *cyclin D1* transcript was significantly reduced in the heat-shocked blastemas of hsDkk1 froglets compared with that in the heat-shocked blastemas of wild-type control froglets (Figure S2). These results support the idea that Wnt/β-catenin signaling was sufficiently inhibited the hsDkk1 froglets.

### Spike regeneration in froglets is sensitive to hsDkk1 after partial denervation of the limb stump

The different responses of tadpoles and froglets to the blockage of Wnt/β-catenin signaling by hsDkk1 expression suggested that their initiation process for limb regeneration also differs. Since *wnt-3a* is expressed in blastemas at both stages, it is unlikely that Wnt/β-catenin signaling is activated only in the tadpole blastema. We therefore thought it was likely that nerve signals were involved in the initiation of limb regeneration and were responsible for the different results following the inhibition of Wnt/β-catenin signaling in tadpoles and froglets. In urodele amphibians, a limb bud does not become heavily innervated until the digital stage of development, and it is not dependent on the nerves for regeneration until this stage [31]. Similar results have been obtained for limb regeneration in *Xenopus* [32]. Therefore, the initiation process of limb regeneration mediated by Wnt/β-catenin signaling might be different in limbs before and after their innervation.

**Figure 6:**
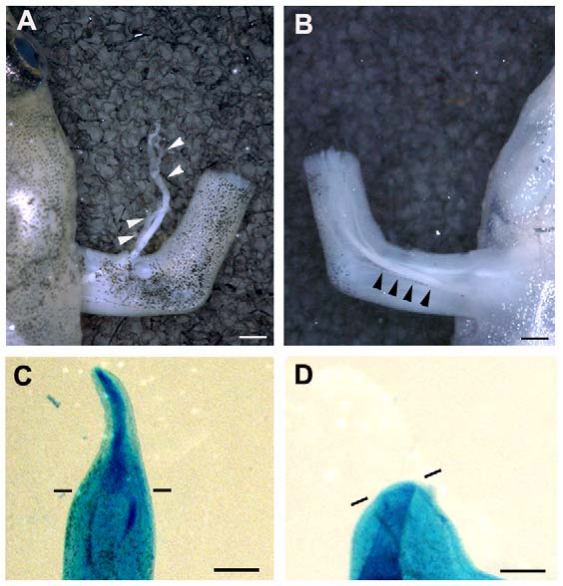
hsDkk1 expression blocked spike regeneration of froglets after partial denervation. (A and B) The dorsal (A) and ventral (B) sides of a froglet forelimb each contain a thick bundle of nerve trunks. The forelimb was amputated through the distal zeugopod level. For observation, an incision was made in the skin on the dorsal side of the shoulder and the nerve trunks were pinched out (A), while nerve trunks can be seen through the intact skin on the ventral side (B). (C and D) After denervation on only the ventral side of a forelimb, the forelimbs of hsDkk1 froglets were amputated, and the animals were repeatedly heat-shocked until 8 dpa. Only one-third of the forelimbs regenerated a spike (C), and most of the forelimbs did not show any regeneration (D). Arrowheads indicate the bundle of nerve trunks. Lines indicate the estimated amputation planes. Scale bar = 500 µm.

We hypothesized that nerve signals, which should be abundant in the limb stump after innervation, can substitute for Wnt/β-catenin signaling during limb regeneration in the froglet. To test this hypothesis, we partially denervated the limb stumps of froglets, since complete denervation prevents limb regeneration in froglets [33], [37] and the effect of blocking the Wnt/β-catenin pathway would therefore be impossible to assess. In limb regeneration of amphibians, nerve requirement for regeneration is quantitative (dependent on the number of axons) and independent of fiber type innervating the limb [16]. The dorsal and ventral sides of a froglet limb each contain a thick bundle of nerve trunks (Figure 6A and 6B). To reduce the nerve signals but not remove them completely, we uprooted the bundle of nerve trunks only on the ventral side and left the nerve trunks intact on the dorsal side, expecting that the nerve signals would be about half of that in the control limbs. Under this condition, we blocked the Wnt/β-catenin signaling by hsDkk1. In the control samples, most of the half-denervated limbs (84.6%; Table 2) still regenerated a spike. Only one-third of the hsDkk1 limbs (33.3%; Table 2) regenerated a spike (Figure 6C), and the remaining 66.7% showed no regeneration at all (Figure 6D; Table 2). Therefore, partial (half) denervation caused the froglet limb stumps to become sensitive to the inhibition of Wnt/β-catenin signaling.

**Figure 7:**
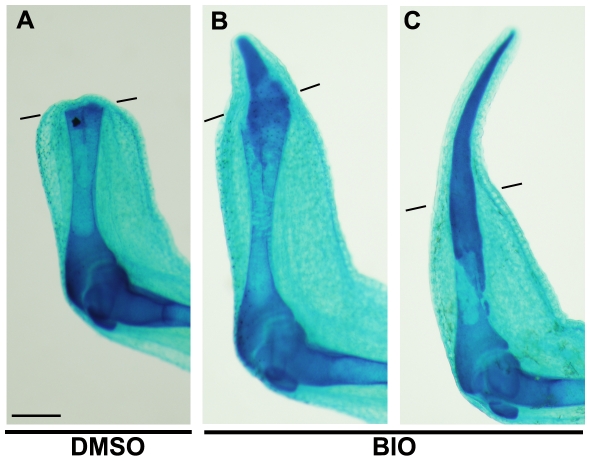
GSK3 inhibitor BIO can rescue spike regeneration of a denervated limb. (A) A DMSO-treated (100 µl/l) control froglet showed no regeneration. (B and C) BIO-treated (1 µM) froglets regenerated a short spike (B) or a long spike (C). Lines indicate the estimated amputation planes. Scale bar = 500 µm.

It is unlikely that the nerve signals were greatly affected by Wnt/β-catenin signaling, because inhibition of Wnt/β-catenin signaling by hsDkk1 did not have the same effect as total denervation in froglets. Rather, the inhibitory effect of hsDkk1 on the half-denervated limbs suggested that the nerve signals and Wnt/β-catenin signaling function redundantly in some aspect of the initiation of froglet limb regeneration and that the role of Wnt/β-catenin signaling might be replaced by nerve signals in the limb stumps without denervation. Conversely, denervation may be rescued by enhancement of Wnt/β-catenin signaling in the initiation of limb regeneration. To test this possibility, we totally denervated limb stumps of froglets and then induced robust activation of Wnt/β-catenin by GSK-3 inhibitor (BIO [48]) treatment. None of 28 forelimbs of denervated froglets reared in DMSO-containing water until 10 dpa were regenerated (Figure 7A; Table 3). Froglets reared in 1 µM BIO-containing water, however, regenerated a spike in 5 (25%) of 20 forelimbs, and 2 of those 5 spikes were relatively long like the spike regenerated from a normal froglet forelimb with nerves (Figure 7B and 7C; Table 3). An exceptional forelimb with DMSO treatment formed a small cartilaginous mass on the stump (Table 3, asterisk), but this structure was clearly shorter than any spike regenerated in BIO-treated froglets (data not sown). Similarly, froglets reared in 0.5 µM BIO regenerated a spike in 5 (29%) of 17 forelimbs, while none of the control froglets with DMSO treatment regenerated a spike (Table 3).

**Table 3:** Regenerative capacity of froglet forelimbs treated with BIO or DMSO.

Type of treatment (BIO or DMSO)	Total number of forelimbs	No regeneration occurred	Some regeneration occurred
		**none**	**1 spike**
**1** µ**M BIO**	20	15	5 (2)
**DMSO (100** µ**l/L)**	28	28*	0 (0)
**0.5** µ**M BIO**	17	12	5 (2)
**DMSO (50** µ**l/L)**	18	18	0 (0)

*One forelimb formed a small cartilaginous mass.

The number in parentheses indicates the number of forelimbs that regenerated a long spike.

## Discussion

### Limb regeneration of a froglet as a model for epimorphosis

In recent years, the involvement of Wnt/β-catenin signaling in animal regeneration has been widely reported across the Metazoa, including in the regeneration of hydra, planarians, and cricket legs [49], [50], [51], [52]. Previous studies also suggested the involvement of this signaling in limb regeneration of *Xenopus* tadpoles [28], [53]. However, it remained unclear whether this signaling is also required for limb regeneration in tadpoles at later stages or in post-metamorphosis froglets. A *Xenopus* froglet can regenerate only a single spike of cartilage after limb amputation, but this regeneration requires the presence of nerves, as does limb regeneration in urodeles [33], [37]. From a functional point of view, this spike regeneration may be an adaptation to help in feeding and mating behavior [54].

Transplant experiments suggest that limb regeneration of froglets resulting in spike formation requires the formation of a wound epidermis and blastema, as in typical epimorphic regeneration rather than a simple tissue (cartilage) regeneration [55]. Recent studies using newly available molecular markers have also indicated that limb regeneration in froglets is epimorphic regeneration (Reviewed in [5]). For example, marker genes for limb regeneration such as *Prx-1*, *Tbx5*, and *Hoxa-13* are expressed in the same patterns in both the froglet limb blastema [33], [37] and axolotl limb blastema [56], [57]. Taking these findings together, we conclude that limb regeneration in froglets is not a simple tissue regeneration but can be a useful model for epimorphic regeneration.

### Different initiation mechanisms of limb regeneration in tadpoles and froglets

A previous study indicated that *wnt-3a*, a Wnt ligand that activates the β-catenin pathway, is expressed in the apical epidermis of regenerating limb buds of *Xenopus* tadpoles [28]. Since *wnt-3a* expression was also detected in the epidermis of the froglet blastema (Figure 1), it is unlikely that the absence of Wnt gene expression in the regenerating froglet limb caused the different results in froglets and tadpoles. Dkk1, a secreted inhibitor of Wnt/β-catenin signaling, interferes with this signaling in a non-competitive manner, regardless of the expression level of *wnt* genes [58], [59]. We therefore think that Wnt/β-catenin signaling is also activated in the froglet blastema and that this signaling activity was efficiently blocked by the Dkk1 transgene used in the present study. It has been shown that Wnt/β-catenin signaling mediates epidermal-mesenchymal interactions during limb formation, inducing *fgf-8* expression in the apical epidermis ([35], [40], [41]; reviewed in [42]). Suppression of *fgf-8* expression in the apical epidermis of blastemas by Dkk1 (Figure 5) indicates that at least this typical function of Wnt/β-catenin signaling for limb formation was sufficiently down-regulated in the hsDkk1 froglets, although we still cannot exclude the possibility that some unidentified activity of Wnt/β-catenin signaling may remain in the blastema.

It is well known that epidermal-mesenchymal interactions are necessary for limb regeneration [60], [61], [62], [63]. In the regenerating blastema, dermis-free epidermis covers the mesenchymal cells, resulting in direct contact between the epidermis and the underlying mesenchyme. In regeneration-incompetent limb stumps, such as denervated limbs, some type of obstruction is often formed between the epidermis and mesenchyme, suggesting that the epidermal-mesenchymal interactions are disrupted. Our histological observations of the dermal layer in the denervated limb stump of the froglet (Figure 4I and 4L; [33], [37]) and the distinct thick basement membrane in the limb bud stump of hsDkk1 tadpoles (Figure 4C and 4E) suggest that the epidermal-mesenchyme interactions are disturbed in such limb stumps. In contrast, no dermal layer or skin glands formed in the blastema of hsDkk1 or wild-type froglets, (Figure 4G, 4H, 4J and 4K), suggesting that the epidermal-mesenchymal interactions were undisturbed in the blastema of hsDkk1 froglets.

Why was there no disruption in the spike regeneration or epidermal-mesenchymal interactions in the froglets by blockage of Wnt/β-catenin signaling? One event that makes a difference in amphibian limb regeneration during ontogeny is innervation of the limb region. Until a limb bud is innervated, its regeneration requires epidermal-mesenchymal interactions but not nerve-derived signals as limb development does. However, nerve axons enter the limb bud at later stages of limb development, and these nerves are thought to wedge between the interacting epidermis and mesenchyme, causing limb regeneration to continue in a nerve-dependent manner [15]. After this transition, the limb regeneration of amphibians will be interrupted if the limb is denervated at a previous stage (medium bud stage) of blastema formation [16]. Conversely, a blastema-like bump can be induced in urodeles if a nerve is deviated to a wound on the side of a limb [17], [18], [19].

Nerves apparently emit some signals that promote the initiation of limb regeneration, and the signals are apparently not associated with Wnt/β-catenin signaling, since the expression of hsDkk1 in froglets never exerted the same effect as denervation. Singer (1952) reported that nerve signals function in a quantitative manner, independent of the fiber type innervating the limb [16]. Thus, the amount of nerve signaling after half-denervation (Figure 6) is probably about half that of a normal limb. We also observed that the expression level of *fgf-8* was decreased by the hsDkk1 transgene expression in froglets (Figure 5B-E). Notably, Wnt/β-catenin signaling can mediate the epidermal-mesenchymal interactions by controlling *fgf-8* expression in a specialized apical epidermis in regenerating limb buds of the *Xenopus* tadpole [28] and in developing amniote limb buds ([35], [40], [41]; reviewed in [42]).

**Figure 8:**
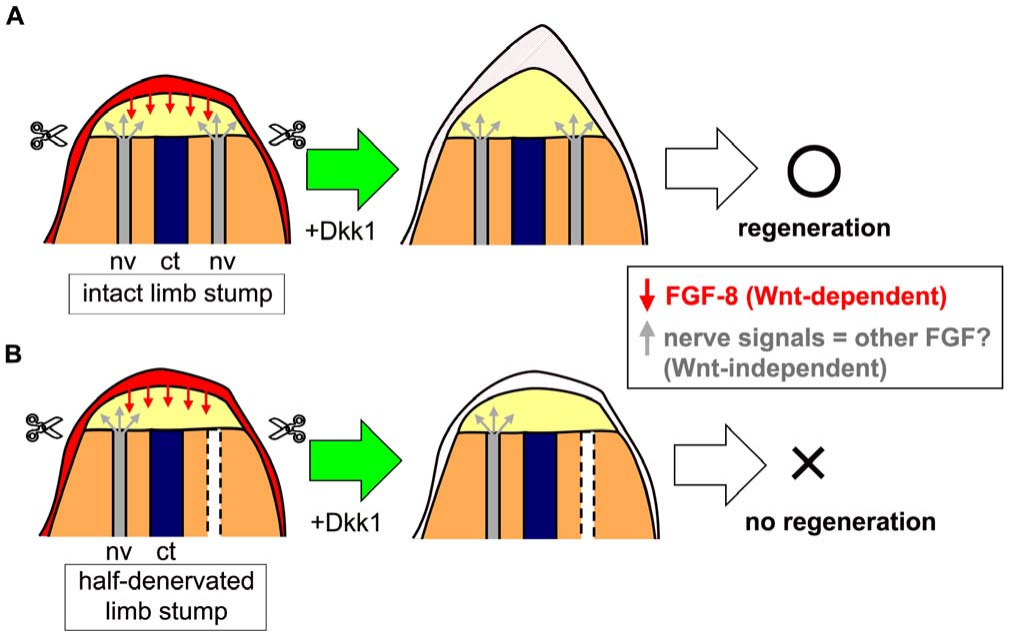
Model for Wnt/*β*-catenin and nerve signals in early limb regeneration in *Xenopus* froglets. (A) Successful blastema formation in the intact forelimb of the hsDkk1 froglet. Wnt/β-catenin signaling (presumably mediated by *wnt-3a* in the epidermis) induces *fgf-8* expression in the epidermis. Induction of Dkk1GFP expression by heatshock diminishes epidermal *fgf-8* expression. However, nerve signals released from the nerves around the limb stump have some redundant function with FGF-8 and/or the product(s) of another Wnt downstream gene(s), whereas Dkk1GFP does not markedly affect the function of the nerve signals. The nerve signals can substitute for the role of the Wnt downstream gene, so that the limb stump can continue the blastema formation process and form a spike, even after blockage of Wnt/β-catenin signaling. (B) Interrupted blastema formation in the half-denervated forelimb of an hsDkk1 froglet. When the limb stump is partially denervated, the amount of nerve signals is thought to be around half of that in an intact limb stump. This level of nerve signals is insufficient to substitute for the role of the Wnt downstream gene. Thus, most of the limb stumps cannot continue the blastema formation process, resulting in no regeneration, after blockage of Wnt/β-catenin signaling. Considering the redundant function of the nerve signals and a Wnt downstream gene, the nerve signals may be mediated by another member of the FGFs. Scissors indicate the amputation plane. nv, nerves; ct, cartilage.

Our findings suggest that nerve signals can functionally substitute for the role of Wnt/β-catenin signaling in the froglet blastema, while the molecule responsible for the nerve signals is independent of the β-catenin signaling pathway (Figure 8). The observation that hsDkk1 exerted an inhibitory effect on spike regeneration of froglets when the nerve signals were reduced to about half (Figure 6) supports this idea. While our data do not directly reveal the molecule responsible for the nerve signals in froglet limbs, the molecule may be associated with a member of the FGF family (Figure 8). Since the nerve signals presumably compensate for the attenuated Wnt/β-catenin signaling, by filling the role of a Wnt/β-catenin-dependent gene(s), it seems possible that the nerve signals involve some member of the FGF family that can substitute for FGF-8 as a downstream effecter of Wnt/*β*-catenin signaling. Results of previous studies indicating FGF-2 as a candidate for the nerve signals in axolotl and *Xenopus* [20], [64] support this hypothesis. However, this hypothesis does not exclude the possibility that other candidates for nerve signals are involved in the initiation of limb regeneration, since nerve signals can consist of a ‘cocktail’ of several different molecules. It is noteworthy that total denervation of the limb stump is sufficient to block limb regeneration of froglets but that simple inhibition of Wnt/*β*-catenin signaling is not sufficient to block the regeneration. Therefore, nerve signals seem to have more significant roles, qualitatively and/or quantitatively, than Wnt/*β*-catenin signaling in the initiation of froglet limb regeneration. In fact, denervation concomitant with limb amputation causes reduction in the expression of multiple genes including *Prx-1*, *Tbx5, msx-1, fgf-8, and fgf-10* in the froglet blastema at 7 dpa [37]. In contrast, a previous work showed that hsDkk1 expression specifically suppressed *fgf-8* expression in the tadpole blastema as early as 8 hours after a heatshock, while expression of *fgf-10*, *msx-2*, *Hoxa-13* and *Lmx-1* was unaffected [28]. Similarly, although hsDkk1 expression suppressed *fgf-8* expression in the froglet blastema within 16 hours after a heatshock (Figure 5B–E), spike regeneration itself was unaffected. These facts suggest that *fgf-8* expression is more directly regulated by Wnt/*β*-catenin signaling than by nerve signals, while both hsDkk1 expression and denervation down-regulate *fgf-8* expression in the froglet blastema. Nerve signals have a more significant role(s) in the initiation of froglet limb regeneration probably because nerve signals can activate not only common target genes that can be activated by Wnt/*β*-catenin signaling but also other target genes implicated in the initiation of limb regeneration. Alternatively, nerve signals may be quantitatively more robust than Wnt/*β*-catenin signaling in activating common downstream targets. If nerve signals and Wnt/*β*-catenin signaling largely overlap in their downstream functions in limb regeneration, robust activation of Wnt/*β*-catenin, for example, by a GSK-3 inhibitor (e.g., BIO [48]) treatment may rescue regeneration of a denervated limb. Alternatively, a large amount of exogenously applied FGF-8 protein as a downstream effecter of Wnt/*β*-catenin signaling may rescue regeneration of a denervated limb. We have actually tested one of these possibilities. GSK-3 inihibitor (BIO) treatment rescued spike regeneration of denervated limbs to some extent (Figure 7; Table 3), and this result strongly supports the hypothesis that nerve signals and Wnt/*β*-catnin overlap in downstream functions in the initiation of limb regeneration.

### Wnt/β-catenin signaling is important in the regeneration associated with epidermal-mesenchymal interactions but may be dispensable for that of a heavily innervated limb

As mentioned above, the results of transplant experiments, nerve dependency, and molecular markers for limb regeneration support the idea that limb regeneration in froglets that results in spike formation is a kind of epimorphic regeneration rather than a simple tissue regeneration (reviewed in [5]). The repression of *fgf-8* expression in the apical epidermis by the induction of hsDkk1 expression (Figure 5B–E) suggests that Wnt/β-catenin signaling also functions in the limb regeneration of a metamorphosed froglet through epidermal-mesenchymal interactions. However, in contrast to the absolute requirement for Wnt/β-catenin signaling for limb regeneration in the tadpole [28], the signaling is less essential for limb regeneration in the froglet, since the froglet can regenerate a spike even if Wnt/β-catenin signaling is inhibited during blastema formation. Considering that hsDkk expression could block the spike regeneration of froglets after partial denervation, Wnt/β-catenin signaling can still function in limb regeneration in the froglet, but it becomes less essential than that in the tadpole. Instead, nerve signals seem to have a more important role for regeneration of the froglet limb (Figure 8). A previous study showed that total denervation of the froglet forelimb stump leads to ectopic apoptosis in blastema cells at 4 dpa and reduced proliferative activity of blastema cells at 7 dpa [37]. These results suggest that total denervation excluded blastema cells at the early stage of limb regeneration by apoptosis, resulting the reduced cell proliferation at a later stage of regeneration and finally in the failure of spike regeneration. Since partial denervation with the hsDkk1 expression also resulted in failure of spike regeneration in froglets, similar ectopic apoptosis in blastema cells at the early stage of regeneration may be induced after partial denervation with inhibition of Wnt signaling.

Regarding the requirement of Wnt/β-catenin signaling in limb regeneration in adulthood, Kawakami et al. (2006) reported that the limb regeneration capacity of adult axolotls is decreased by infection of the limb stump with an adenovirus carrying a Wnt antagonist (Axin-1) [53]. It is noteworthy that this infection did not completely eliminate regeneration but resulted in hypomorphic spike-like regeneration (3 of 5 samples, 60%; [53]). Thus, Wnt/β-catenin signaling may be dispensable at least for the hypomorphic regeneration of an adult limb that has been heavily innervated. Kawakami et al. (2006) also transferred an adenovirus carrying Dkk-1 into amputated limb buds of larval axolotls and observed complete blockage of regeneration in a few of the limb stumps (2 of 26 samples, 7%; [53]). The limb buds of the larval axolotl may not have been heavily innervated, thus making it possible for Dkk-1 expression to completely block limb regeneration in a few samples. The results obtained by Axin-1-adenovirus infection suggested that Wnt/β-catenin signaling is required for regeneration of a complete limb in adulthood. However, with the current sample size, it is difficult to be conclusive about how essential the signaling is. Further studies are required to elucidate the role of Wnt/β-catenin signaling in complete regeneration of an adult limb that has been heavily innervated.

In recent years, transgenic protocols for urodele amphibians have become available [65], [66]. Thus, the gene manipulation in limb regeneration performed in the anuran amphibian *Xenopus* in this study should also be feasible in urodele amphibians, enabling direct comparison of the regeneration mechanisms in these organisms. In such experiments, it would be intriguing to compare the regenerative responses of urodele limbs before and after heavy innervation, when Wnt/β-catenin signaling is inhibited during the initiation of limb regeneration. It would also be interesting to perform such a gene manipulation during limb regeneration of urodele amphibians in combination with partial denervation, as we performed in *Xenopus* froglets. If our observations in *Xenopus* can be extended to urodele amphibians, it would provide more general insights into the mechanisms of initiation of vertebrate limb regeneration. Since the early steps of regeneration are critical for determining the extent of the regenerative response after injury, such elucidation could lead to new strategies for organ-level replacement therapies in the future.

## Materials and Methods

### Ethical treatment of animals

The law (Act on Welfare and Management of Animals) in Japan exempts study using *Xenopus laevis* (amphibians) from requiring IRB approval. All surgery was performed under ethyl-3-aminobenzoate anesthesia, and all efforts were made to minimize suffering.

### Animal husbandry

*Xenopus laevis* adults and froglets were obtained from domestic animal vendors. The tadpoles and froglets were kept in dechlorinated tap water at 21–23°C. The *Xenopus* tadpoles were staged according to Nieuwkoop and Faber [36]. The containers were cleaned daily, and the tadpoles were fed powdered barley grass (Odani Kokufun Co., Ltd., Kouchi, Japan). At stage 58, the feeding was stopped until metamorphosis was completed. After metamorphosis, the froglets were fed tubifex every other day.

### Reverse transcription-PCR

Total RNA samples were extracted from blastemas using the TRIzol® Reagent (Invitrogen), purified through spin columns of the RNA mini kit (Qiagen), and reverse-transcribed with the Transcriptor First Strand cDNA Synthesis Kit (Roche) using oligo(dT) primers, according to each manufacturer's instructions.

Primers specific for *Xenopus laevis wnt-3a* (forward primer, 5′- GGAGATTATGCCGAGCGTA-3′; reverse primer, 5′- GGCTGACTCTCTTGTGGCTTTA-3′), *cyclin D1* (forward primer, 5′-CAACGCCTCACACTTTTCCT-3′; reverse primer, 5′-TTGTGTTGCTGCTGTGCTTG-3′), and *ribosomal L8* (forward primer, 5′-GTGGTGTGGCTATGAATCCT-3′; reverse primer, 5′-ACGAGCAGCAATAAGACCAACT-3′) were used. These primers were designed to include intronic sequences to avoid amplifying genomic DNA. For the RT-PCR of *wnt-3a*, amplification of *ribosomal L8* was used as a loading control, because *ribosomal L8* mRNA level remains relatively constant during development [67]. The cycle conditions were 94°C for 2 min, 30 cycles at 94°C for 15 s, 54.3°C for 30 s, and 72°C for 30 s, and then a final extension at 72°C for 5 min. The PCR products were detected on ethidium bromide-stained agarose gels. Real-time quantitative PCRs of *cyclin D1* were carried out using a Light Cycler and the SYBR Green Labeling System (Roche) with the following cycling protocol: a 95°C denaturation step for 10 minutes followed by 40 cycles of denaturation at 95°C (10 s), annealing at 60°C (10 s), and extension at 72°C (6 s). The fluorescent product was detected at the end of a 72°C extension period. Gene expression was normalized to that of *ribosomal L8*. The PCR products were subjected to a melting curve analysis, and the data were analyzed and quantified using Light Cycler software. The results are shown as values relative to the expression level observed in wild-type froglet blastemas. In Figure S2, the level in wild-type was defined as 1.0. The quantification was performed four times using total RNA derived from four independent samples.

### DNA constructs and in situ hybridization

The tdTomato-poly(A) cassette was inserted downstream of the *Xenopus γ-crystallin* promoter [68]. The IS-crystallin-tdTomato was generated by introducing the*γ-crystallin* promoter-tdTomato-poly(A) cassette into ISceI-pBS II SK+ [69]. The hsp70-Dkk1GFP-crystallin-tdTomato for transgenesis was generated by introducing the hsp70–Dkk1GFP5-poly(A) cassette excised from hsp70-Dkk1GFP [28] into the IS-crystallin-tdTomato (Figure 2A).

Dig-labeled RNA probes of *wnt-3a* [70] and *fgf-8* [71] were prepared according to the protocol of the manufacturer (Roche). To prepare serial cryosections, the specimens were fixed in MEMFA, embedded in OCT compound (Sakura), and serially sectioned at 10 µm in thickness. Transcripts were detected by in situ hybridization on frozen sections using the procedures described by Yoshida et al. [72].

### Transgenesis in *Xenopus laevis*

Transgenic *Xenopus laevis* embryos were generated by a modified REMI technique using oocyte extract instead of egg extract ([73], [74]; reviewed in [75]). To minimize leakiness of the transgene under the *hsp70* promoter, the embryos were reared at 16°C in 0.1X MBS [76]. After they started swimming and feeding, the tadpoles and froglets were reared at 22–23°C, like the non-transgenic individuals.

For heat-shocking, the tadpoles or froglets were placed in 34°C water for 30 min, as described by Beck et al. [24]. Three to four hours after heat-shocking, the F0 individuals were examined under a fluorescence dissecting microscope and classified as GFP-positive (hsDkk1GFP) or GFP-negative (wild-type). The tadpoles and froglets were examined again the next day to confirm their GFP fluorescence. F0 individuals with mosaic expression patterns of GFP and ambiguous individuals that did not show GFP fluorescence 3 to 4 hours after heat-shocking but showed weak GFP the next day were excluded from the experiment. To guarantee correct comparisons, GFP-positive (hsDkk1GFP) and GFP-negative (wild-type) F0 individuals were treated in exactly the same way in a series of experiments.

### Tadpole and froglet surgery

Tadpoles and froglets were anesthetized in 1∶5000 ethyl-3-aminobenzoate (Tokyo Chemical Industry) dissolved in Holtfreter's solution. The tadpole hindlimb buds were amputated at the presumptive knee level (according to the outside view and a fate map by Tschumi [77]) with an ophthalmologic scalpel. The tadpole forelimb buds can be seen in a cavity (forelimb atrium) situated ventral to the posterior portion of the pronephros and dorsal to the gill region [36]. After heat-shocking, a tiny hole was made on the roof skin of the forelimb atrium with a 30G injection needle, and the skin was torn with two sharp forceps from this hole. Note that this process tears the skin sealing the forelimb atrium but not the skin of the limb bud itself. The forelimb buds were then amputated at the presumptive elbow level (according to the outside view and reported expression patterns of *Hoxa11* [78] and *Sox9* [39]. After metamorphosis was completed, the cartilage pattern of the areas of the amputated limbs was examined under a dissecting microscope to evaluate the limb regeneration. The froglet forelimbs were amputated through the distal zeugopodium with ophthalmologic forceps, and the amputation surface was trimmed to be flat. Denervation of the amputated limbs was performed according to the method of Endo et al. [33]. To judge their regenerative capacity, the froglets were kept for at least one month for regeneration after limb amputation.

In some cases, the limbs were stained with Alcian blue, as described previously [71].

### BIO treatment

A 1 mM stock solution of GSK3 inhibitor IX (BIO; Calbiochem) dissolved in DMSO was stored in the dark at 4°C. Froglets soon after forelimb amputation and total denervation were raised in dechlorinated tap water with BIO solution (experimental) or with the same amount of solvent, DMSO (control), until 10 dpa. BIO- or DMSO-containing water was changed every other day. Since BIO is a light-sensitive compound, the containers including water and froglets were kept in the dark during BIO or DMSO treatment until 10 dpa.

### Histology

The limb stumps and blastemas of tadpoles and froglets were excised and fixed in Bouin's fixative. The specimens were then dehydrated and embedded in paraffin. Sections were cut at a thickness of 6 µm and stained with hematoxylin, eosin, and Alcian blue, using standard procedures.

## Supporting Information

Figure S1 **hsDkk1 inhibited the forelimb bud regeneration in tadpoles.** (A and B) Dorsal view of the left forelimb bud at stage 54. A paddle-shaped forelimb bud can be seen in a cavity dorsal to the posterior portion of the gill region (A). The hsDkk1GFP expression was induced in the entire tadpole body, including the forelimb bud region, by heat-shock (B). (C and D) Dorsal view of the left forelimb of a froglet after amputation at stage 54. The forelimb bud regeneration was inhibited in the hsDkk1 tg individual (C), while a complete forelimb with four digits was regenerated in the wild-type control (D). Lines indicate the estimated amputation planes. ante, anterior; post, posterior; prox, proximal; dist, distal. Scale Bar = 250 µm for (A) and (B), and 1 mm for (C) and (D).
(TIF)

Figure S2 **Effect of hsDkk1 expression on the transcript abundance of** ***cyclin D1*** **in the froglet blastema.** The gene expression level was measured by real-time PCR using specific primers. The results were first normalized to *ribosomal L8* and then represented as a value relative to the *cyclin D1* expression level in the blastemas of wild-type control froglets. The quantification was performed four times using the total RNA derived from four independent samples. The value represents the mean of four independent experiments, with standard error. Asterisk indicates the change was statistically significant (*P<0.05) by Student's *t-*test.
(TIF)

Table S1 **Regenerative capacity of tadpole forelimb buds heat-shocked and amputated at stage 54.**
(DOC)
