# Supplementary figures and images for: Different Requirement for Wnt/β-Catenin Signaling in Limb Regeneration of Larval and Adult Xenopus
Source: PLoS One. 2011 Jul 26;6(7):e21721. doi: 10.1371/journal.pone.0021721 (PMC3144201; doi:10.1371/journal.pone.0021721)

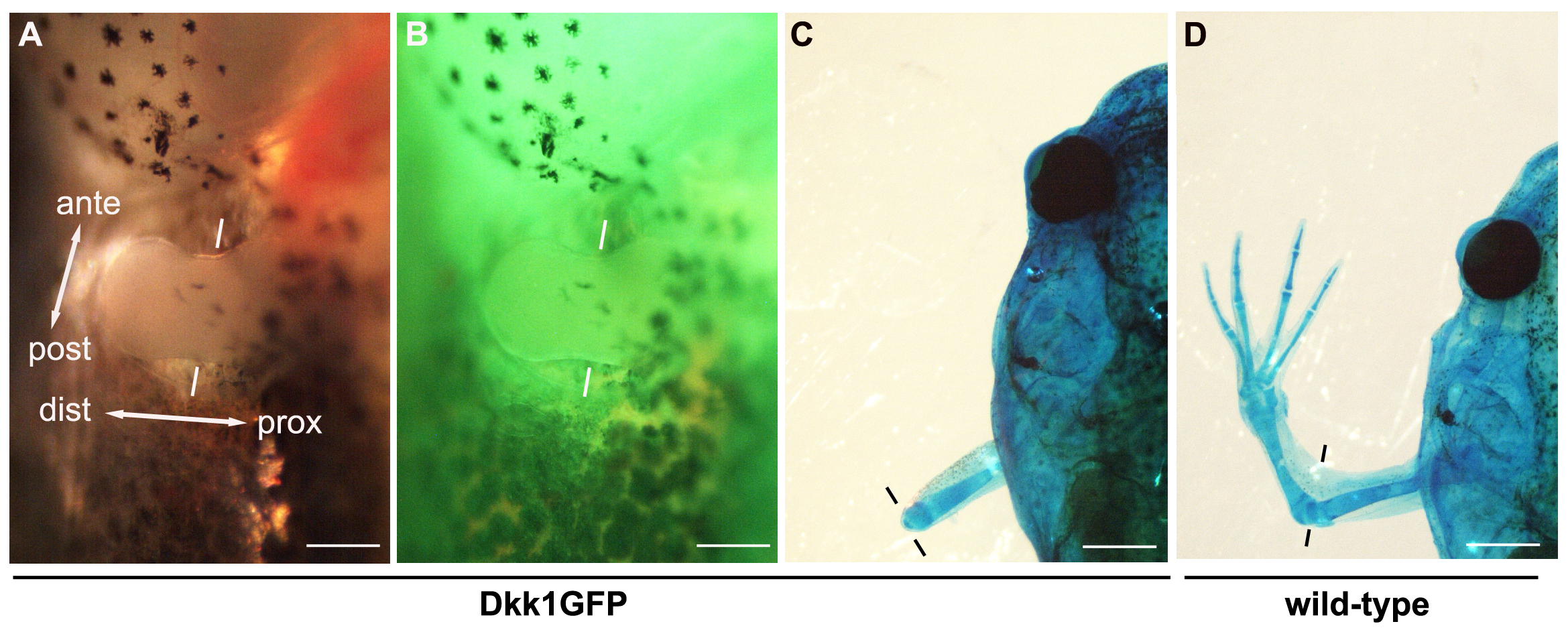

Supplement: Figure S1 — hsDkk1 inhibited the forelimb bud regeneration in tadpoles. (A and B) Dorsal view of the left forelimb bud at stage 54. A paddle-shaped forelimb bud can be seen in a cavity dorsal to the posterior portion of the gill region (A). The hsDkk1GFP expression was induced in the entire tadpole body, including the forelimb bud region, by heat-shock (B). (C and D) Dorsal view of the left forelimb of a froglet after amputation at stage 54. The forelimb bud regeneration was inhibited in the hsDkk1 tg individual (C), while a complete forelimb with four digits was regenerated in the wild-type control (D). Lines indicate the estimated amputation planes. ante, anterior; post, posterior; prox, proximal; dist, distal. Scale Bar = 250 µm for (A) and (B), and 1 mm for (C) and (D). (TIF) [file pone.0021721.s001.tif]

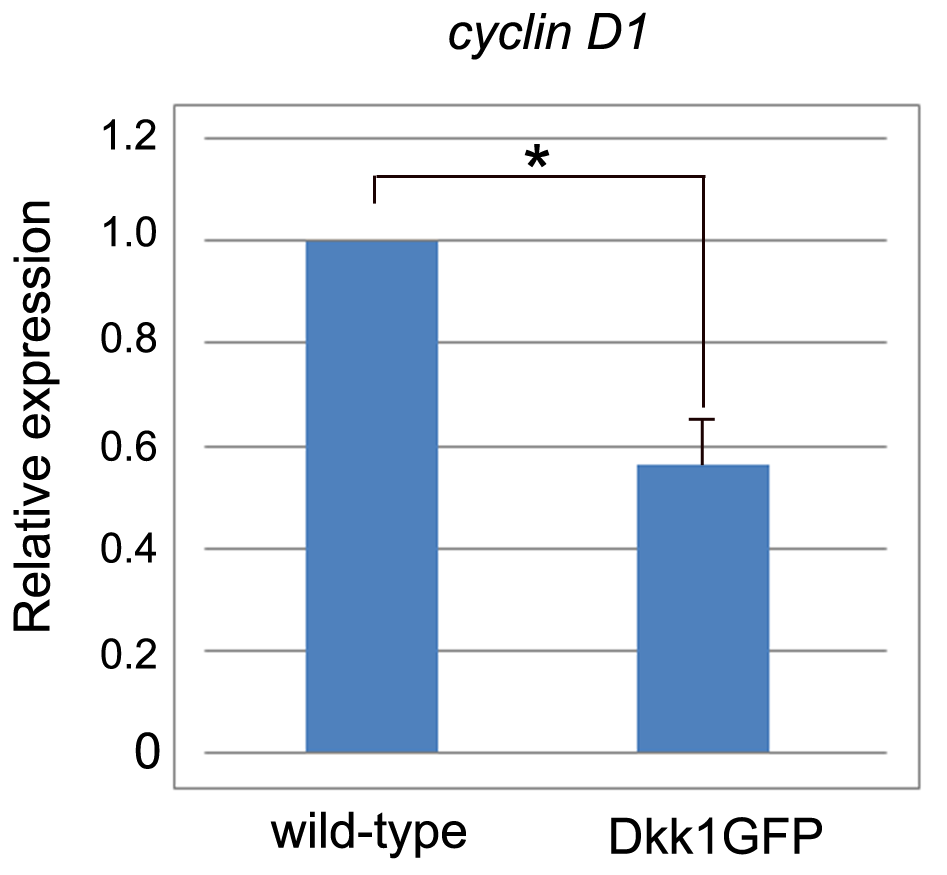

Supplement: Figure S2 — Effect of hsDkk1 expression on the transcript abundance of cyclin D1 in the froglet blastema. The gene expression level was measured by real-time PCR using specific primers. The results were first normalized to ribosomal L8 and then represented as a value relative to the cyclin D1 expression level in the blastemas of wild-type control froglets. The quantification was performed four times using the total RNA derived from four independent samples. The value represents the mean of four independent experiments, with standard error. Asterisk indicates the change was statistically significant (*P<0.05) by Student's t-test. (TIF) [file pone.0021721.s002.tif]
