# Supplementary material for: Different Requirement for Wnt/β-Catenin Signaling in Limb Regeneration of Larval and Adult Xenopus
Source: PLoS One. 2011 Jul 26;6(7):e21721. doi: 10.1371/journal.pone.0021721 (PMC3144201; doi:10.1371/journal.pone.0021721)
Supplement: Table S1 — Regenerative capacity of tadpole forelimb buds heat-shocked and amputated at stage 54. (DOC) [file pone.0021721.s003.doc]

**Table S1**

**Regenerative capacity of tadpole forelimb buds heat-shocked and amputated at stage 54**

| **Types of tadpole (wild-type or hsDkk1)** | **Total number of limb buds** | **No regeneration occurred** | **Some regeneration occurred** | | | | |
| --- | --- | --- | --- | --- | --- | --- | --- |
|  |  |  | **Incomplete<----------------------------------------------->** | | | | **complete** |
|  |  | **none** | **1 spike** | **1 digit** | **2 digits** | **3 digits** | **4 digits** |
| **wild-type** | 14 | 1 | 1 | 3 | 0 | 0 | 9 |
| **Dkk1GFP** | 11 | 9 | 0 | 2 | 0 | 0 | 0 |
